# Establishment of the National Technology Validation and Implementation Collaborative (NTVIC) and Forensic Investigative Genetic Genealogy Technology Validation Working Group (FIGG-TVWG)

**DOI:** 10.1016/j.fsisyn.2023.100317

**Published:** 2023-01-25

**Authors:** Matthew J. Gamette, Ray A. Wickenheiser

**Affiliations:** Idaho State Police Forensic Services, Meridian, ID, USA; New York State Police Crime Laboratory System, Albany, NY, USA

## 1 Establishment of the National Technology Validation and Implementation Collaborative

The National Technology Validation and Implementation Collaborative (NTVIC) was established in 2022 with a vision to collaborate nationally on validation, method development, and implementation. The group is not associated with any organization, corporation, or non-profit. However, the group has connections with prominent forensic science membership organizations, universities, and private technology and research companies. The top leaders of the NTVIC Steering Group are federal, state and large local laboratory directors. The founding group of state and large local laboratory directors share a common vision to share existing resources to work together on these validation and implementation projects to lessen the burden on individual forensic science and forensic medicine providers to perform the work. Currently, forensic providers often duplicate validation studies, method development, or performance verification out of necessity. Forensic science service providers have learned that conducting these studies collaboratively has several benefits: 1) The most informed and brightest minds in the country can be used to generate plans, procedures and troubleshoot issues. 2) The existing resources at laboratories can be pooled to implement the solution more quickly and provide more robust data sets. 3) The validation work can be published as a collaborative effort for purposes of public scrutiny. 4) The developmental validation work product can culminate in a technology transfer and implementation plan for instrument purchase, performance verification, and quick implementation in other laboratories interested in the technology. Founding members of the NTVIC are:

• Idaho State Police Forensic Services (Matthew Gamette—Chair)
• California Department of Justice Bureau of Forensic Services (Barry Miller)
• Colorado Bureau of Investigation Forensic Services (Lance Allen)
• Department of Defense DNA Operations for the Armed Force Medical Examiner Service (Dr. Timothy McMahon)
• Kentucky State Police Forensic Science Laboratory (Laura Sudkamp)
• Miami-Dade Police Department, Forensic Services Division (Stephanie Stoiloff)
• Minnesota Bureau of Criminal Apprehension Forensic Science Services (Catherine Knutson)
• Missouri Highway Patrol Crime Laboratory Division (Brian Hoey)
• New York City Office of Chief Medical Examiner's Office (Timothy Kupferschmid)
• New York State Police Crime Laboratory System (Dr. Ray Wickenheiser)
• Ohio Bureau of Criminal Investigation (Roger Davis)
• Texas Department of Public Safety (Brady Mills)
• Wisconsin Department of Justice Division of Forensic Sciences (Jennifer Naugle)

## 2 Mission

The mission of the National Technology Validation and Implementation Collaborative (NTVIC) is to share resources and strategies to rapidly implement technology and new methods into publicly funded forensic science service provider (FSSP) and forensic science medical provider (FSMP) facilities in a scientifically sound and defensible manner.

## 3 Vision

The National Technology Validation and Implementation Collaborative is a group of visionary publicly funded FSSPs and FSMPs with a shared desire to perform developmental or other validation of new instruments or methods and facilitate their implementation. The NTVIC invites participation from publicly funded FSSPs and FSMPs, federal government agencies and laboratories, vendors, researchers and technology experts, and others interested in validation and implementation of technology or new methods in publicly funded forensic science laboratories. A NTVIC steering group of forensic science laboratory directors, leaders, researchers, and educators determines projects that the collective team will collaborate on to developmentally or otherwise validate with a focus on sharing the responsibility and workload for validation planning, analytical work, documentation, and peer reviewed publication of findings.

Committees will be established for each specific project with interested member participants and contributors. Members will select committees and subcommittees to lead and participate in based on interest, need, resources, and other considerations. Each project committee will address elements of training for forensic science providers, policy development, and technical considerations such as appropriate instrumentation, procedures, and validation required. Each committee formed will solicit appropriate collaborators and partners, determine the contributions needed from each participant, secure funding necessary to accomplish validation, oversee the technical aspects of robust validation and implementation, and ensure that an implementation plan is published for other forensic science providers to implement. The focus of each subcommittee will be validation for participants, creation of an implementation plan for other interested laboratories, and publication of work in peer-reviewed journals. All publications will emphasize best laboratory and quality management processes.

## 4 Purposes

• To increase the speed of technology and method validation and implementation into publicly funded FSSPs and FSMPs
• To share available validation resources at publicly funded forensic science facilities for faster implementation
• To plan, accomplish, and publish scientifically sound and defensible validation studies in peer reviewed publications
• To contribute to standardization of technology implementation in public forensic science entities, utilizing the latest standards
• To facilitate the establishment of technology specific working groups to support continuous improvement of validated and implemented technology
• To publish, after validation, a performance verification implementation plan for other laboratories, complete with purchasing information, appropriate methods, and performance verification plan
• To provide a not-for-profit home for federal funding that supports technology and method implementation
• To provide a venue for early adopter forensic science providers to interact and collaborate
• To ensure each technology, instrument, or method is implemented with appropriate policy, procedure, and quality management

The National Technology Validation and Implementation Collaborative members sign a Memorandum of Agreement to participate in good faith and contribute resources to the collaborative. While this group functions independently of any forensic science organization, private company, or government entity, this group has integrated with these organizations to share information and ensure project goals are achieved.

## 5 First NTVIC Technology Validation Working Group

The first Technology Validation Working Group of the NTVIC is focused on the implementation of Forensic Investigative Genetic Genealogy (FIGG), Forensic Genetic Genealogy (FGG), and Investigative Genetic Genealogy (IGG) in public law enforcement agencies and forensic science laboratories. Over twelve Forensic Laboratory Directors from state or major regional laboratories convened this working group of the NTVIC to investigate transitioning the lab work and also genealogical searching from strictly the private laboratory space into the public forensic science laboratory space. While this group will help establish validation standards in support of the public laboratories that have purchased instrumentation and are navigating implementation of single nucleotide polymorphism (SNP) based technology, the focus of this group will be robust SNP array or sequencing based approaches, with a focus on WGS combined with genealogical searching and building of family trees to develop investigative leads. The working group has engaged with several major research institutions, private companies currently working in the space, federal agencies and laboratories, and top-tier researchers. The short-term goals of the committee are to:

1) Train public forensic science practitioners and law enforcement investigators to perform genealogical searching;
2) Develop a national network of trained public forensic practitioners that can work as a team to solve difficult cases and teach other public forensic science professionals to use the technology;
3) Develop robust policy and procedures for states to immediately implement the responsible use of this technique and technology;
4) Determine and standardize terminology that will be used in this forensic science discipline;
5) Evaluate the appropriate instrumentation and methods that will be fit for purpose, affordable, and possible for public laboratories to implement in-house for WGS, SNP array testing, or other NGS SNP panels;

The longer-term goals of the committee are to:

1) Purchase and validate the instrumentation and methods and implement WGS and robust SNP array analysis in public forensic science laboratories for investigative purposes;
2) Develop a package of resources such as procurement documentation, analytical methods, performance verification plans, publication templates to implement this technology in other public forensic science laboratories for investigative purposes;
3) Secure funding for this technology to be implemented in public forensic science laboratories;
4) Evaluate the potential viability for SNPs for identity confirmation as an alternative to STR testing for those samples that cannot produce an STR due to degradation, amount of DNA, and other factors.

The FIGG Technology Validation Working Group (TVWG) includes scientists from government and public laboratories, private DNA laboratories, research universities, and technology and instrumentation companies. While this group is overseen by the National Technology Validation and Implementation Collaborative Steering Group, a variety of experts are invited to participate based on expertise and experience with the technology. Four FIGG TVWG subcommittees have been formed to address the training, policy, terminology and technical issues of FIGG. The work of this group, and updates of the progress, will be presented at forensic science and other scientific meetings. The group will focus on peer reviewed publications in scientific journals as the official work products.

**Figure:**
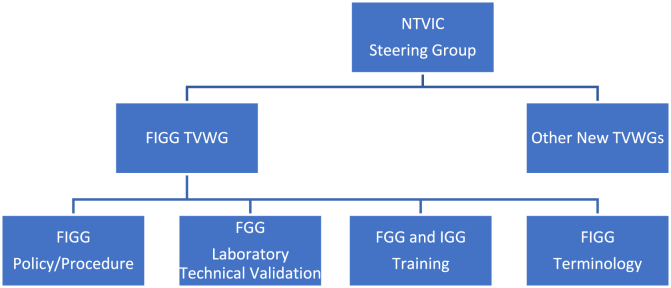

## 6 Important FIGG TVWG disclaimers

• The focus of the FIGG TVWG is for investigative purposes. The FIGG TVWG will consider following SWGDAM validation criteria as appropriate in developmental validation and performance verification recommendations of SNP based analysis for FIGG and DNA based identification.
• The FIGG TVWG has legal advisors from various state agencies and state attorney general offices to guide policy and procedure development, but does not provide legal advice to consumers of Group work products.
• Current participants are volunteers on the FIGG TVWG. While their salaries and travel may be paid by public or private employers, their contributions to the Group are not reimbursed. If any federal government or private funding is used for this work, it will be made publicly transparent to avoid conflict of interest concerns.

Participating members of the FIGG NTVIC are:

• Idaho State Police Forensic Services (Matthew Gamette—FIGG TVWG Chair)
• New York State Police Crime Laboratory System (Dr. Ray Wickenheiser—Subcommittee Chair FIGG Policy/Procedure)
• Center for Human Identification, University of North Texas Health Science Center at Fort Worth (Dr. Mike Coble—Subcommittee Chair FGG Laboratory Technical Validation)
• University of New Haven and Henry C. Lee Institute of Forensic Science (Dr. Claire L. Glynn—Subcommittee Co-Chair FIGG Training)
• Idaho State Police Forensic Services (Rylene Nowlin—Subcommittee Co-Chair FIGG Training)
• Colorado Bureau of Investigation Forensic Services (Lance Allen—Subcommittee Chair FIGG Terminology)
• BODE Technology (Michael Cariola)
• BODE Technology (Sarah Cavanaugh)
• California Department of Justice Bureau of Forensic Services (Barry Miller)
• California Department of Justice Bureau of Forensic Services (Nicola Marie Duda)
• California Department of Justice Bureau of Forensic Services (Daniela Cuenca)
• Center for Human Identification, University of North Texas Health Science Center at Fort Worth (Jonathan King)
• Colorado Bureau of Investigation Forensic Services (Dr. Jennifer Malone)
• Department of Defense DNA Operations for the Armed Force Medical Examiner Service (Dr. Timothy McMahon)
• Armed Forces Medical Examiner System's Armed Forces DNA Identification Laboratory (AFMES-AFDIL) (Charla Marshall)
• Idaho State Police Forensic Services (Rylene Nowlin)
• Idaho State Police Forensic Services (Taylor Maichak)
• Kentucky State Police Forensic Science Laboratory (Laura Sudkamp)
• Miami-Dade Police Department, Forensic Services Division (Stephanie Stoiloff)
• Miami-Dade Police Department, Forensic Services Division (Adriana Kristaly)
• Minnesota Bureau of Criminal Apprehension Forensic Science Services (Catherine Knutson)
• Minnesota Bureau of Criminal Apprehension Forensic Science Services (Ann Marie Gross)
• Missouri Highway Patrol Crime Laboratory Division (Brian Hoey)
• New York City Office of Chief Medical Examiner (Timothy Kupferschmid)
• New York City Office of Chief Medical Examiner (Meredith Rosenberg)
• New York State Police Crime Laboratory System (Julie Pizziketti)
• New York State Police Crime Laboratory System (Urfan Mukhtar)
• Ohio Bureau of Criminal Investigation (Roger Davis)
• Ohio Bureau of Criminal Investigation Laboratory Services Division (Kristen Slaper)
• Othram Inc (Andrew Singer)
• Othram Inc (David Mittelman)
• Signature Science, LLC (Erin Gorden)
• Texas Department of Public Safety (Brady Mills)
• Verogen Corporation (Dr. Swathi A. Kumar)
• Verogen Corporation (Dr. Meredith Turnbough)
• Wisconsin Department of Justice Division of Forensic Sciences (Jennifer Naugle)
• Wisconsin Department of Justice Division of Forensic Sciences (Melisa Wittkowske)

## Declaration of competing interest

The authors declare that they have no known competing financial interests or personal relationships that could have appeared to influence the work reported in this paper.

